# The processing of subthreshold visual temporal order is transitory and motivation-dependent

**DOI:** 10.1038/s41598-023-34392-5

**Published:** 2023-05-11

**Authors:** Patrik Polgári, Ljubica Jovanovic, Virginie van Wassenhove, Anne Giersch

**Affiliations:** 1 University of Strasbourg, INSERM U1114, Strasbourg, France; 2 Laboratoire des Systèmes Perceptifs, École Normale Supérieure, PSL University & CNRS, Paris, France; 3 Cognitive Neuroimaging Unit, CEA, INSERM, CNRS, Neurospin, Université Paris-Saclay, 91191 Gif/Yvette, France; 4 Department of Psychiatry, University Hospital of Strasbourg, 1, Pl de L’Hôpital, 67000 Strasbourg, France

**Keywords:** Human behaviour, Perception

## Abstract

Processing a sequence of events is different from encoding the relative order of the elements composing the sequence. Whether order processing arises automatically from the sequential processing of events is yet unknown, however the literature suggests that order processing can occur at an automatic level when the order of stimuli is not detected consciously. In the present study, we aimed to investigate the question of automatic order processing in a difficult visual task where participants identified one among two possible target luminances. The luminance of the targets was contingent on the order of presentation of two visual cues separated by a subthreshold asynchrony. Participants' performance was compared to that in a control condition where the cues were presented synchronously. In a first experiment, participants’ performance benefited from the use of subthreshold order information compared to the control condition, however this facilitation effect was transient and disappeared over the course of the experiment. In a second experiment, we investigated and confirmed the role of motivation, via a monetary incentive, on the previously observed effect. Taken together, our results suggest that the processing of temporal order of sub-threshold asynchronies is possible, although fragile and likely dependent on task requirements.

## Introduction

Order is an intrinsic property of our conscious experience. We experience time as flowing in a linear fashion (*i.e.*, ‘the arrow of time’) and events, including our percepts, our actions, and even our thoughts, take place either simultaneously or successively on the arrow of time. Thus, ordered sequences are ubiquitous in our environment and our everyday behavior^1^. However, an order of percepts does not necessarily imply a percept of order^2^. Visual events that take place in our environment provide sequential inputs in the brain. Whether this automatically entails the coding of temporal relationships of the different events relative to one another (*i.e.*, processing the order of the events) is still an open question in psychology and cognitive sciences. Does the perception of order arise automatically from the sequential processing of events? Or is perceiving order derived only in constrained conditions when additional cognitive processes are mobilized?

From an empirical standpoint, an automatic processing of order is plausible in light of several experimental results suggesting that the brain can process temporally ordered sequences. When the order of the stimuli composing sequences is clearly visible, humans can extract and encode sequences implicitly, *i.e.*, when task instructions do not draw attention to the fixed temporal structure of the stimulus presentation, or when participants are unable to report the previously viewed sequences in a post-test phase^3–6^. For example, in one study^3^ four consecutive supraliminally presented stimuli appear sequentially in different locations, and the participants have to react after each stimulus. Participants’ performance benefits from the repetition of a given sequence pattern, even when participants are not informed and do not detect any pattern^3^. Furthermore, automatically encoded sequences can be exploited implicitly to facilitate the detection of a subsequent target by guiding attention to its location^7,8^. Studies in uni- and multi-sensory perception showed that the sequential order in which stimuli are presented is also encoded implicitly as a temporal sequence^9^. Additionally, implicit sequences of stimuli can influence the detection of the synchrony between the stimuli on subsequent trials when the sequences are presented supraliminally (*i.e.,* the asynchrony between elements of the sequence can be detected) or at a subthreshold level (*i.e.,* asynchrony under the threshold of conscious perception)^10–13^. These results suggest that the brain can process temporally ordered sequences in an automatic manner.

Nevertheless, processing the sequence of stimuli is not equivalent to the processing of the relative position of the individual stimuli inside the sequence, that is, ordering them. Processing a sequence of stimuli, or benefitting from the repetition of a sequence, can be based on an automatic serial preparation to successive stimuli, *i.e.,* an automatic replay of the sequence^14^. It does not require the coding of a higher-level representation of order between successive elements. The formation of such a representation might take unnecessary time which would be inconsistent with the high temporal resolution of automatic, unconscious mechanisms allowing us to follow information fluently over time^13^. Moreover, automatically deriving the order of any event relative to one another would mean a computational overload for the brain when one is in a busy street full of dynamic events that are not necessarily related to one another. This is consistent with recent findings of brain responses suggesting that intermodal temporal order may not be coded automatically^15^. Given these arguments it is reasonable to speculate that temporal order is not processed automatically for all events, but only when order processing bears a benefit which outweighs the cost of such automatic processing.

In a recent study, Chassignolle et al.^16^ found evidence suggesting that the order of stimuli that is not detected consciously can still be processed at an automatic level. In their cued reaction time task paradigm, the order of two colored cues (‘red then green’ or ‘green then red’) predicted the upcoming target shape (‘ + ’ or ‘x’) to which participants responded manually. Participants were informed about the contingency between cue order and targets prior to taking part in the experiment. Participants improved their reaction times (RT) when the ordered cues were separated by suprathreshold, easily detectable 66 ms asynchronies, but also and, importantly, by subthreshold, undetected 17 ms asynchronies, compared to a control condition where the cues appeared synchronously (*i.e*., no physical order information). These results indicate that, even when under the threshold of conscious perception, order can be processed and used to prepare a response. However, the effect sizes were rather small, and participants were informed about the relationship between the order of the cues and the target. We sought to explore further the observed effects, by exploring their robustness across time and the influence of motivation.

In the present study we aimed to replicate the facilitation effect linked to an automatic order processing described by Chassignolle et al.^16^ We used a more difficult task than in Chassignolle et al.^16^, to verify whether order could be used to orient participants’ response choice in addition to speeding up their responses. Like in Chassignolle et al.'s study, the paradigm consisted in verifying whether subjects could use a short 17 ms asynchrony between two visual cues to predict target luminances. Participants identified target luminances, and those were contingent on the order of presentation of the two visual cues. Importantly, in order to replicate the conditions of Chassignolle et al.’s study, participants were explicitly told which subthreshold order cue was followed by which target luminance. As a matter of fact, preliminary studies without this instruction did not yield any effect of subthreshold temporal cues. Thus, even though in this study we used the most favorable conditions for the effect to emerge, we did not expect large effects of subthreshold order cues.

In our first experiment, the order of the cues predicted the targets luminances in one group (‘Predictive’ group), while in another group contingency was removed after training so that the order of the cues did not predict the upcoming luminance during the task (‘Non-predictive’ group). Our design was aimed to verify whether the asynchrony could have a non-specific arousing effect, independent of any order effect. Any non-specific effect of the asynchrony between the cues was expected to result in improved performance in both groups for asynchronous trials, whereas a facilitation effect that is specifically linked to the automatic processing of order information should lead to improved target detection only in the ‘Predictive’ group, and possibly a deterioration in the ‘Non-predictive’ group when contingencies were removed after training. Furthermore, we had a long enough task to verify the evolution of performance over time. We reasoned that if participants’ responses were based on the learning of the temporal sequence of cues and targets, similar to a temporal contextual cuing effect (*e.g.*, “left cue” *then* “right cue” *then* “light gray target”), performance would stabilize and even improve over time asymptotically^3–5^. In contrast, if the subthreshold order information can indeed be exploited, resulting from the formation of a higher-level representation of order and its use to predict the target (*e.g.*, “left first, right second” *so* “light gray target”), it is likely transitory rather than stable. As mentioned above, stimuli always follow each other in quick succession in the environment, and not all stimulus sequences are meaningful. In fact, most of them are meaningless. If any succession of signals was to yield the formation of a stable representation of order, it would mean an exponential computational cost for the brain. If the formation of higher-level representation of order exists, it is likely flexible and transitory, occurring only when the benefit linked to the formation of an order representation outweighs the computational costs. Considering that our task is difficult because of the lack of conscious confirmation of the order information with sub-threshold asynchronies and the low discriminability of the target luminances, we may expect a degradation of participants’ performance at a distance of the training phase^17^.

We conducted a second experiment to explore more directly if the effects were under endogenous control, and more specifically modulated by participants’ motivation. The same design was used as for the ‘Predictive’ group in Experiment 1 for everyone, but this time participants’ motivation was manipulated via a monetary incentive in two groups (‘Incentivized’ vs. ‘Non-incentivized’ groups). A stronger improvement in luminance identification that is linked to the order of the cues in the Incentivized group compared to the Non-incentivized group would suggest that motivation can play a role in the modulation of the use of subthreshold order information.

## Experiment 1

### Methods

#### Participants

Twenty-five healthy participants (20 females/5 males, mean age ± SD = 21.16 ± 2.19) were recruited for Experiment 1. All participants had normal or corrected-to-normal vision and reported no neurological or psychiatric disorders. After analyzing performances in the Temporal order judgment (TOJ) tasks, one participant was excluded from further analyses because of above chance performance for 17 ms asynchronies. Following Chassignolle et al.^16^ , in the TOJ tasks, individual performances for 17 ms SOA trials were compared to chance level performance using a χ^2^ test. Our TOJ tasks comprised 40 SOA = 17 ms trials (20 with left–right, 20 with right-left order), and the threshold above which performance was considered as above chance corresponded to 29 correct detections out of 40 trials. We considered that order with 17 ms stimulus onset asynchrony (SOA) was not subthreshold for the excluded participant. Responses in the No-Go condition were low, ranging between 0 and 7, with most participants having a score of 0 or close to 0. Thus, no selection was done based on No-Go trials. Further analyses were conducted on the remaining 24 participants (19 females/5 males, mean age ± SD = 21.17 ± 2.24).

The project was approved by the local ethics committee of the University of Strasbourg (Unistra/CER/2018–02/3). All participants gave their informed written consent in accordance with the declaration of Helsinki (2018) and received a monetary compensation of 15 euros.

#### Equipment and apparatus

The experiment was conducted in a quiet, dimly lit room. The experimental paradigms were run using the MATLAB software (R2007a, MathWorks) with CRS VSG Toolbox for MATLAB. Stimuli were generated on a 20″ 120 Hz CRT screen (resolution 800 × 600) using ViSaGe (Visual Stimulus Generator) by Cambridge Research Systems. Manual responses were recorded using two response-buttons. The distance between participants and the screen was maintained fixed at 110 cm using a chin rest.

#### Stimuli

The background was set to a gray (21.0 cd/m^2^) color throughout the experiment. All trials started with a central fixation dot surrounded by two black squares (0.76 cd/m^2^, side length 0.7 deg VA) on the left- and right-hand side. The distance between the centers of the two squares was set to 4.8 degrees of visual angle. Each square contained at its center a dot identical to the central fixation dot. After a fixed period, the dots inside the squares turned white (47.5 cd/m^2^) representing the cues which followed three spatiotemporal patterns corresponding to the three conditions that were intermixed and run in the Experiment (Asynchronous, Synchronous, No-Go, see Experimental tasks and procedure section). Targets were two gray values that were designed to differ only slightly in luminance (light gray 18.3 cd/m^2^, dark gray 16.7 cd/m^2^) so that they would be difficult to distinguish. Targets were chosen to be difficult to identify in order to avoid ceiling effects in performance that would mask a potential facilitation effect. With low enough baseline correct response rates a facilitation effect was possible to be observed if subthreshold order information was processed automatically and exploited.

The distance and the delay between the flash cues were chosen so that apparent motion is unlikely to take place^18^. We wanted to avoid a perception of apparent motion because it might have facilitated the detection of order^19,20^.

#### Experimental tasks and procedure

##### Subthreshold temporal order cued luminance identification (subTOLI) task

The task consisted of three experimental conditions and trials of each condition were intermixed and presented in a randomized order within the subTOLI task.

**Figure 1:**
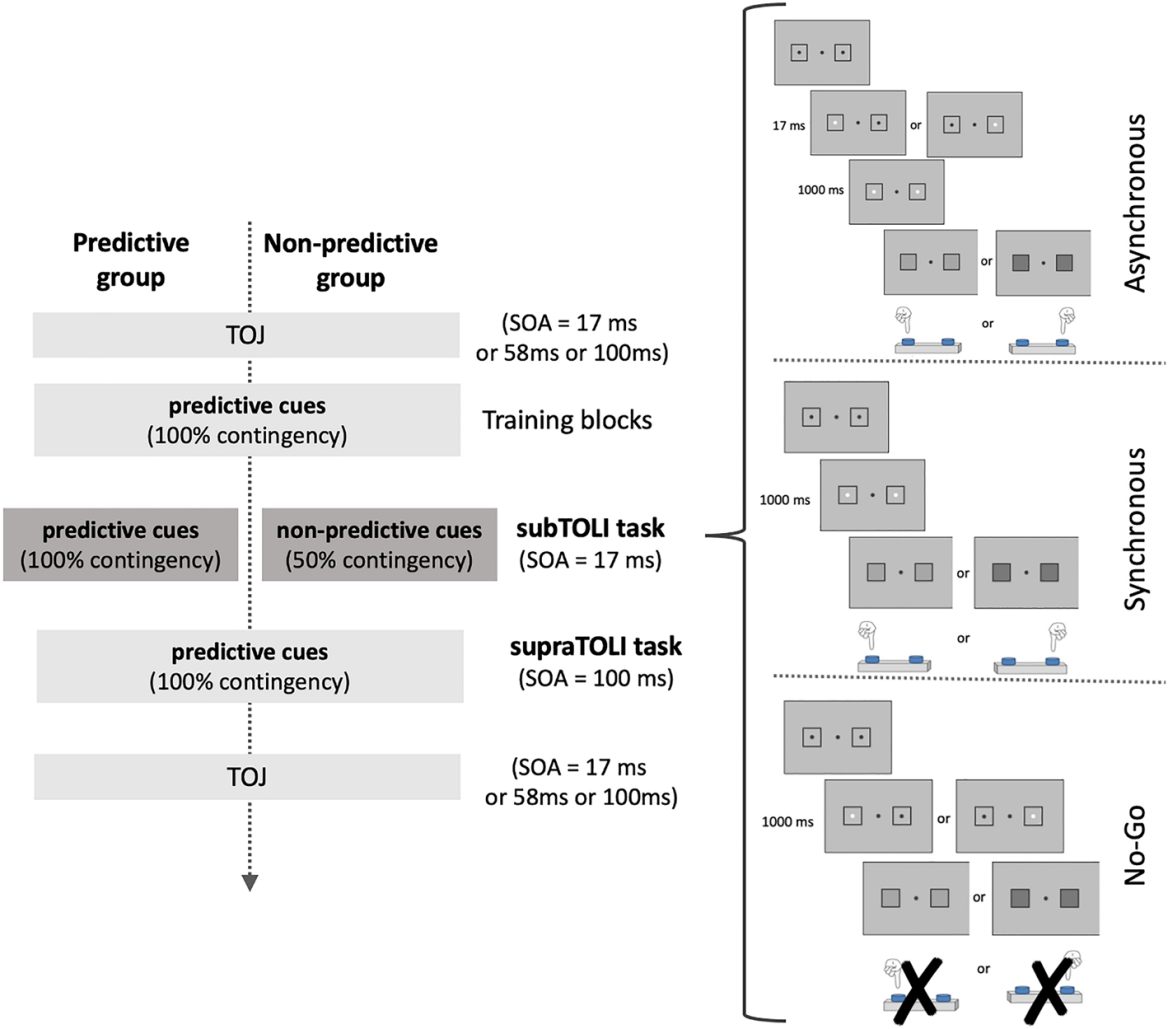
Schematic representation of the stimuli used in the subTOLI task and procedure of Experiment 1. All trials started with a central fixation dot surrounded by two squares, each containing a dot. Cues corresponded to the two lateral dots changing color from black to white either one after the other, separated by a 17 ms delay (right upper panel), or synchronously (right middle panel). After a 1000 ms presentation period the cues were replaced by the target luminances (‘light gray’ or ‘dark gray’) that participants had to identify via a button press on the corresponding side (left or right). Note that the difference between the target luminances is exaggerated on the figure for illustration purposes, in the actual task the difference between light and dark gray targets was significantly less noticeable. In the No-Go condition (right bottom panel) the cue corresponded to only one of the dots turning white for 1000 ms, after which the target appeared. In this No-Go condition participants were instructed to withhold their response. In the example shown in the figure, a ‘left then right’ cue order is associated to the ‘light gray’ target and a leftward response, while a ‘right then left’ cue order is associated to the ‘dark gray’ target and a rightward response. Trials of the three conditions were mixed and displayed in a randomized order in the tasks. The association between cue order, target luminance and response side were all counterbalanced between participants. Stimuli and timeline are not drawn to scale. The design of Experiment 1 (left panel) involved two groups that differed only in the contingency rate between the order of the asynchronous cues in the subthreshold temporal order cued luminance identification (‘subTOLI’) task, with the ordered cues always predicting the identity of the targets in the Predictive group and the cues bearing no predictive value for the Non-predictive group. Importantly, the Non-predictive group was instructed the same way as the Predictive group and told that the contingency rate was 100% (for the contingency they were previously trained on). Both groups began and ended the experiment with a temporal order judgment (‘TOJ’) task with three SOAs (17 ms, 58 ms, 100 ms). The groups performed their respective version of the subTOLI task (dark boxes), then the same suprathreshold version of the temporal order cued luminance identification (supraTOLI) task with the ordered cues predicting the identity of the targets in 100% of the Asynchronous trials.

In the Asynchronous condition (Fig. 1, right top), the cues (*i.e.*, black dots inside the squares turning white) appeared one after the other, separated by a 17 ms asynchrony. Each cue order (‘left–right’ and ‘right-left’) was equally represented. The cues remained on the screen for 1000 ms, and were then replaced by a target (*i.e.*, one of two gray values) filling the squares on both sides synchronously. The target remained on the screen until a response was given by the participant via a left or right button-press.

The Synchronous condition (Fig. 1, right middle) was identical to the Asynchronous condition except for the white cues which appeared synchronously on the screen (SOA = 0 ms).

The No-Go control condition (Fig. 1, right bottom) was identical to the Asynchronous condition with the difference that only one of the two lateral dots turned white for a duration of 1000 ms before the presentation of the target gray value. In this condition, participants were instructed not to make a button-press in response to the gray value. This condition was introduced to motivate participants to attend to both the first and second cue before responding to the target in the other two experimental conditions. Theoretically, it would be possible to predict the upcoming target by attending only to the position of the first or the last element of the sequence of the cue stimuli, without taking into account the order relationship between them. For instance, if participants attended only to the first stimulus, this spatial cue would be enough to predict the identity of the target (*e.g.*, ‘left’ so ‘light gray’). Since response is to be inhibited during No-Go trials, participants are forced to attend to the whole cueing sequence. A high commission error rate in the No-Go condition would indicate that the participants did not take into account both of the cues before giving a response in the other two conditions, thus it was used as an exclusion criterion.

The task consisted of two blocks of equal length separated by a short break. Each block consisted of 48 Asynchronous, 48 Synchronous, and 24 No-Go trials that were intermixed, yielding a total of 2 × (48 + 48 + 24) = 240 trials per subTOLI task per participant.

Two groups were tested in the experiment; each group was distinguished by different cue order-target contingency rates in the Asynchronous trials of the subTOLI task, *i.e.,* the subthreshold temporal order cued luminance identification (but the instructions in the subTOLI task and the other tasks of the experiment did not differ for the two groups). The experimental design and procedure are depicted in Fig. 1 (left panel, “subTOLI task”). In the Predictive group (n = 12, 11 females) the luminance of the target gray value (light or dark) was contingent on the order of the cues in the Asynchronous condition (‘left–right’ or ‘right-left’), thus the subthreshold order predicted the upcoming target in 100% of the trials. The combinations of cue order (‘left–right’ or ‘right-left’), target gray value (light or dark) and response side (left or right) were counterbalanced between participants in this group.

In the Non-predictive group (n = 12, 8 females), a given cue order preceded one target gray value in only 50% of the trials and the other target in the remaining 50% of the trials, thus the subthreshold order did not predict the upcoming target. The combinations of target gray value and response side were counterbalanced between participants in this group. The Non-predictive group was introduced in the experimental design to make sure that any facilitation effect observed on participants’ performance in the Asynchronous condition compared to the Synchronous condition specifically resulted from the processing of the order of the cues and not from a non-specific effect of the asynchrony between the cues. The two groups did not differ in age [F(1,22) = 0.29, p = 0.59, partial η^2^ = 0.01].

Prior to performing the subTOLI task, all participants completed four training blocks. The aim of this training was for participants to associate one subthreshold cue order with one target luminance, so that e.g., “left then right” cue signaled a “light gray” target and “right then left” signaled a “dark gray” target. The first training block, used to familiarize participants to the target gray values, consisted of 24 randomly presented light and dark gray trials where participants received oral feedback on the identity of each target gray value and were asked to press the response-buttons accordingly. In the second training block, both the Predictive and Non-predictive groups were told about the contingency between the subthreshold temporal order of the cues and the target gray values and performed two sub-blocks of 30 trials, each containing only light gray or dark gray trials (with the corresponding cue orders), in a randomized order. The aim of this training block was to promote the association between each subthreshold order and the corresponding target gray value (similar to a classic conditioning paradigm). In the third training block participants performed 20 Asynchronous trials with the two types of asynchronies (cue order with its associated gray level) in randomized order and in the fourth training block a short, 40 trial-long version of the subTOLI task (16 Asynchronous, 16 Synchronous, and 8 No-Go trials intermixed and presented in a randomized order). All participants received the same explicit instructions about the specific association between the subthreshold order of the cues and the target gray values present in the task and were trained the same way in the four training blocks. For the Non-predictive group, the association remained true in only 50% of the Asynchronous trials in the main subTOLI task, however, participants were not informed that the association became invalid after training (Fig. 1). This was done so that both groups attend to and try to use similarly the subthreshold order cues. If subthreshold order cues can be used to predict the upcoming targets, an improvement in target discrimination should be observed in the Asynchronous condition compared to the Synchronous condition in the Predictive group. In contrast, in the Non-predictive group, the subthreshold order cues should lead to errors in 50% of the trials, if the cues are used in the same way as during the training; in other words, we expect impaired performance in the Asynchronous compared to the Synchronous condition in the Non-predictive group. Hence the comparison of performance between the two groups should be the most sensitive measure to evidence an impact of the subthreshold order cues.

##### Suprathreshold temporal order cued luminance identification (supraTOLI) task

This task was performed as a control after the subthreshold temporal order cued luminance identification task in order to check that both groups were able to use an order cue to predict the upcoming target gray value when order is suprathreshold and when it predicts the target in 100% of the Asynchronous trials. This time, both groups performed the same suprathreshold task (*i.e.*, 100% contingency in Asynchronous trials) (Fig. 1), meaning that the cues were predictive even in the so-called ‘Non-predictive’ group.

This task was identical to the subTOLI task except for the asynchrony in the Asynchronous condition being 100 ms (and thus easily detectable) instead of 17 ms, and the shorter task length (24 Asynchronous, 24 Synchronous and 12 No-Go intermixed trials presented in a randomized order in one single block, yielding a total of 60 trials).

Similar facilitation effects in both the Predictive and Non-predictive groups when order is presented with 100 ms SOA and predicts the target in 100% of the trials would confirm that there is no difference between the groups in their ability to process suprathreshold order and use it to predict the gray level of the target. This would also mean that the potential difference in performance between the two groups in the subthreshold version of the task would likely be due to the manipulation of the proportion of trials where the order of the cues correctly predicts the target.

This task was preceded by two training blocks. The first one contained 30 Asynchronous trials and the second a 30 trial-long version of the task (with intermixed Asynchronous, Synchronous and No-Go trials).

##### Temporal order judgment (TOJ) tasks

At the beginning and at the end of the protocol, participants performed a classical TOJ task (Fig. 1). Stimuli were identical to the cues in the previously described tasks but this time, the target luminances were not presented.

Three SOAs separating the onset of the white dots were used (17 ms, 58 ms, and 100 ms), each presented over 40 trials in a randomized order, with equal numbers of ‘left–right’ and ‘right-left’ order presentations, yielding a total of 3 × 40 = 120 trials per TOJ task. Participants were instructed to make a button-press on the side of the first stimulus. This task was used to make sure that participants did not detect order with 17 ms SOA at the beginning or the end of the experiment, so that order remained subthreshold in the main task of the experiment.

#### Statistical analysis

We report repeated measures ANOVAs and differences were localized using sub-analyses. D-prime values, that index luminance identification abilities, and beta (or criterion), reflecting a bias to respond “light gray” or “dark gray”^21^, were calculated using the psycho Package^22^ in the R Studio environment^23^. All statistical analyses were carried out on the Statistica® software. The level of significance was set to α = 0.05 throughout the analyses. The partial eta-squared (η^2^) was added as a measure of effect size.

### Results of Experiment 1

The ability to identify the gray values was quantified by calculating d-prime for each participant in each block (four sub-blocks in the subthreshold, one main block in the suprathreshold version of the task) and each condition (Asynchronous vs. Synchronous). For this, we arbitrarily considered correct responses to light gray targets as hits and correct responses to dark gray targets as correct rejections. Thus, incorrect responses to light gray targets were considered misses and incorrect responses to dark gray targets were considered as false alarms.

#### SubTOLI task

**Figure 2:**
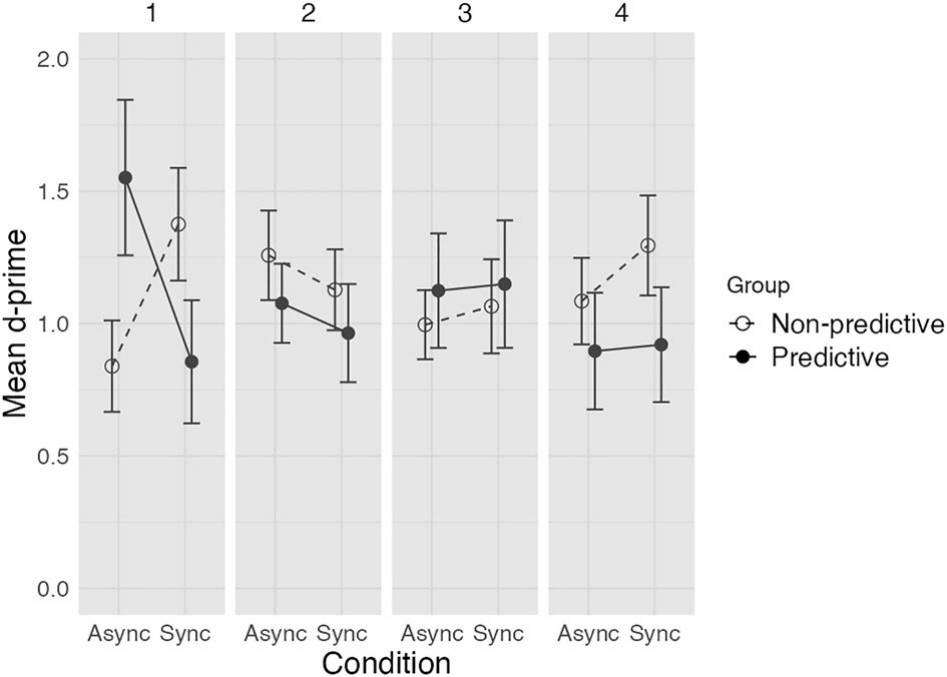
Mean d-prime values of the two experimental groups (Predictive vs. Non-predictive) in the Asynchronous (‘Async’) and Synchronous (‘Sync’) conditions in the 4 sub-blocks of the subthreshold temporal order cued luminance identification task. In the subthreshold version of the task the contingency between order cues and targets was manipulated between the two groups (the order cues predicted the target in 100% of the trials in the Predictive group and in 50% of the trials in the Non-predictive group). Error bars represent ± SEM between participants.

We divided the trials into four sub-blocks to investigate the evolution of performance over time, and to test for a potential improvement or deterioration of performance. The binning of our dataset was a compromise between the number of trials necessary for meaningful statistical results (24 Asynchronous vs. 24 Synchronous trials / sub-block) and the observation of a potential transitory effect upon a preliminary visual inspection of our dataset. We compared sub-blocks with equal number of trials in each condition (first set of 24 Asynchronous and 24 Synchronous trials in the 1st sub-block, second set in the 2nd sub-block, etc*.*). A repeated measures ANOVA on d-prime with condition (Asynchronous vs. Synchronous) and sub-block (1st vs. 2nd vs. 3rd vs. 4th) as within-group variables, and group (Predictive vs. Non-predictive) as a between-group variable revealed a significant three-way interaction [F(3,66) = 3.99, p < 0.05, partial η^2^ = 0.15] (Fig. 2).

We calculated for each participant the difference in performance between the Asynchronous and Synchronous conditions (*i.e.*, d’_async_ – d’_sync_) in each sub-block as a proxy for the facilitation effect linked to the use of subthreshold temporal order information. We then conducted ANOVAs on this value in each sub-block with group as a between-group variable. A significant effect of group was found only in the 1st sub-block [F(1,22) = 14.10, p < 0.005, partial η^2^ = 0.39] with a larger positive performance difference in the Predictive group (0.70) compared to the Non-predictive group (-0.54). In the other sub-blocks, no significant group effects were found (Fig. 2).

We further compared d-prime between groups in each condition (Synchronous and Asynchronous) and each sub-block. This analysis was conducted to verify that the group effect is related to the Asynchronous rather than the Synchronous condition. In the Asynchronous condition of the 1st sub-block a significant effect of group was found [F(1,22) = 4.37, p < 0.05, partial η^2^ = 0.17] with a higher d-prime in the Predictive group (1.55) compared to the Non-predictive group (0.84). No other group effects were found in the other sub-blocks.

In order to verify whether the experimental manipulation also affected participants’ decision criterion in addition to d-prime, we conducted a repeated measures ANOVA on the decision criterion with condition (Asynchronous vs. Synchronous) and sub-block (1st vs. 2nd vs. 3rd vs. 4th) as within-group variables, and group (Predictive vs. Non-predictive) as a between-group variable. No significant main effects or interaction between factors was revealed.

Additionally, we analyzed subthreshold order detection sensitivities in the subTOLI and the TOJ tasks. Results were in line with our main findings showing higher subthreshold order discrimination sensitivity in the subTOLI task compared to the TOJ task in the Predictive group. This difference became significant in the Non-predictive group when only taking into account trials that were congruent with training (contingency between order and luminance level staying the same between the training and the main experiment). The detailed analyses can be found in Supplementary material S2.

#### SupraTOLI task

An ANOVA conducted on d-prime with condition (Asynchronous vs. Synchronous) as a within-group variable and previous group distinction (Predictive vs. Non-predictive) as a between-group variable revealed a main effect of condition [F(1,22) = 20.98, p < 0.0005, partial η^2^ = 0.49], with a higher d-prime value in the Asynchronous condition (2.17) compared to the Synchronous condition (1.20). No main effect of group or interaction between factors was found, indicating similar d-prime values in both groups. As a reminder, these results were expected since the cues were predictive in both groups in the supraTOLI task, even in the so-called ‘Non-predictive’ group.

An ANOVA on the decision criterion with the same factors revealed no significant main effects or interactions between factors.

### Discussion of Experiment 1

The results suggest that the subthreshold temporal order of the cues was processed and used to predict targets, thus improving luminance identification performance, but only for a very short period at the beginning of the task. This initial pattern of results replicates the facilitation effect observed on RT by Chassignolle et al.^16^ in their study and extends it to participants’ accuracy.

The facilitation effect observed in the suprathreshold version of the task in the two groups indicates that the groups did not differ in their general ability to detect and use order to predict an upcoming target. Hence, the results observed with the subthreshold cue order are unlikely due to a difference between the groups, and more likely to our experimental manipulation, namely the contingency between the subthreshold order of the cues and the target gray values introduced for the Predictive group. The observed pattern is consistent with the transient formation and exploitation of an order representation to predict targets.

Although present at the beginning of the task (1st sub-block), the facilitation effect linked to the cue order-target contingency is transitory and the effect disappears by the 2nd sub-block. This result indicates that the effect of the exploitation of subthreshold order is rather small. The transiency and fragility of the facilitation effect suggest that the effects do not result from a purely automatic sequential learning process. Given the difficulty of the task, with very small differences between the target gray values, it might be possible that motivation plays a role. Participants can be expected to be more motivated at the beginning than at the end of a monotonous and difficult task^24,25^. Experiment 2 was designed to verify this hypothesis by manipulating participants’ motivational level via a monetary incentive.

## Experiment 2

### Materials and methods

#### Participants

Thirty-five healthy participants (25 females/10 males, mean age ± SD = 23.34 ± 2.67) were recruited. All participants had normal or corrected-to-normal vision and reported no neurological or psychiatric disorders. After analyzing performances in the TOJ tasks, 5 participants were excluded from further analyses with the same criteria as in Experiment 1, because of above chance performance for 17 ms asynchronies and thus we considered that order with 17 ms SOA was not subthreshold for these subjects. Responses in the No-Go condition were low, ranging between 0 and 11, with most participants having a score of 0 or close to 0. Thus, no selection was done based on No-Go trials. Further analyses were conducted on the remaining 30 participants (22 females/8 males, mean age ± SD = 23.47 ± 2.61).

We conducted a power analysis in order to verify the sample size required to replicate the effects of Experiment 1 (power of 0.83, alpha error probability of 0.0125) (as a reference we took the analysis comparing d’_async_ – d’_sync_ between the two groups). The required number of participants (24 in total, 12 per group) was well below the actual number of participants used in Experiment 2 (30 in total, 15 per group).

The project was approved by the local ethics committee of the University of Strasbourg (Unistra/CER/2019–08). All participants gave their informed written consent in accordance with the declaration of Helsinki. In this experiment participants’ motivation was manipulated via a monetary incentive. Thus, at recruitment participants were promised 15 € and one group (Incentivized group) was proposed a doubling of their monetary compensation after the first part of the subTOLI task, if they improved their performance in the second part. This group was composed of 15 participants (11 females) and the other group (Non-incentivized) was also composed of 15 participants (11 females). The two groups did not differ in age (Table 1). For ethical reasons, after completing the experiment, all participants received a monetary compensation of 30 euros regardless the group they belonged to or their performance at the task.

**Table 1:** Average age and average questionnaire sub-scale scores in the experimental groups.

	Incentivized group average score	Non- incentivized group average score	F	*p *value	partial η^2^
age (years)	22.60	24.33	F(1,28) = 3.61	0.07	0.11
DSSQ ‘success motivation’	17.23	19.87	F(1,26) = 2.39	0.13	0.08
DSSQ ‘intrinsic motivation’	28.54	28.07	F(1,26) = 0.18	0.68	0.007
DSSQ ‘overall motivation’	4.31	4.07	F(1,26) = 1.37	0.25	0.05
BAS ‘reward responsiveness’	15.80	17.60	F(1,28) = 4.00	0.055	0.13
BAS ‘drive’	8.73	8.33	F(1,28) = 0.36	0.56	0.01
BAS ‘fun seeking’	10.67	11.20	F(1,28) = 0.55	0.46	0.02
BIS	20.00	21.73	F(1,28) = 1.82	0.19	0.06

#### Equipment and apparatus

The same experimental setting was used as described in Experiment 1.

#### Experimental task and procedure

**Figure 3:**
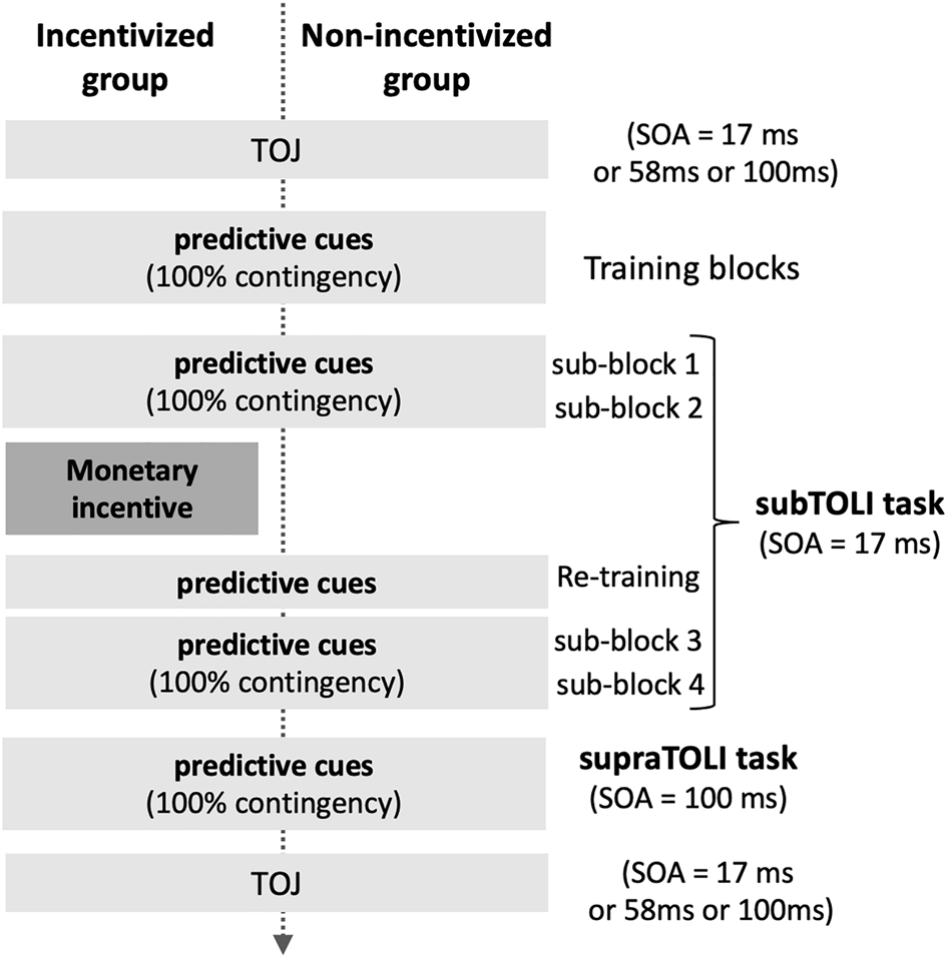
Procedure of Experiment 2. In Experiment 2 the two groups differed by the introduction of a monetary incentive in the middle of the subTOLI task. The Incentivized group received instructions motivating them to attend to and exploit the order of the cues to improve their performance, while the Non-incentivized group received no incentive and carried on the second block of the task as before. Importantly, in Experiment 2 both groups performed the same tasks, with the ordered cues predicting the identity of the targets in 100% of the Asynchronous trials in both the subTOLI and supraTOLI tasks.

The same experimental protocol and tasks were used as for the Predictive group in Experiment 1 with 3 differences. The experimental design and procedure are schematized in Fig. 3.

(i) Unlike in Experiment 1, cue order-target contingencies existed in 100% of Asynchronous trials for both groups. There were four sub-blocks of 24 trials in the Asynchronous, 24 trials in the Synchronous, and 12 trials in the No-Go condition intermixed and presented in a randomized order within a sub-block, yielding a total of 4 × (24 + 24 + 12) = 240 trials. The main structure of the experiment was the same as in Experiment 1 (*i.e.*, two blocks separated by a break, each block containing two sub-blocks).
(ii) Participants were divided into two groups. The Incentivized group received a monetary incentive after the first block of the subTOLI task with the instructions specifying that if their performance in the identification of the target gray values improved in the second block of the task their monetary compensation would be doubled. After these instructions and before starting the second block of the task, they performed again the Conditioning training block. The Non-incentivized group only performed the Conditioning training block between the two blocks of the subTOLI task and received no additional instructions. The re-training with the conditioning training block was designed to increase the chance that subjects would use the order to identify the target gray values. Since the motivational instructions were introduced in only one of the groups, the two blocks of the task had to be distinguished in the analyses (*i.e.,* before vs. after motivational instructions).
(iii) Before starting the experimental tasks all participants completed the French versions of the Dundee Stress Scale Questionnaire (DSSQ)^26^ and the BIS/BAS scale^27^. The DSSQ was used to evaluate participants’ baseline motivational levels (*i.e.*, before the experiment) with three sub-scores: ‘success motivation’ measuring their motivation to excel in their performance, ‘intrinsic motivation’ measuring their interest in the task, and their ‘overall motivation’. With the BIS/BAS scale we were interested in the ‘reward responsiveness’ subscale because we hypothesized that, in combination with the monetary incentive, this trait may interact with participants’ motivational levels and performance.

#### Statistical analysis

In addition to the statistical analyses described in Experiment 1 we also performed ANCOVAs on data from the subTOLI task.

### Results of Experiment 2

#### Questionnaire scores

Separate ANOVAs with group as a between-group variable conducted on the DSSQ subscales indicated that the Incentivized and Non-incentivized participants had similar scores for all sub-scales of the questionnaire (Table 1).

A similar analysis was used to compare the groups on their score on the BIS/BAS ‘reward responsiveness’ sub-scale. The difference between groups was not significant, however a tendency was found with a slightly higher score in the Non-incentivized group (17.60) compared to the Incentivized group (15.80) (Table 1). We hypothesized that the ‘reward responsiveness’ trait may potentially interact with participants’ motivation and performance in the detection of subthreshold order following the monetary incentive. For this reason, we computed the improvement of detecting subthreshold order for each individual over the course of the experiment as the difference between an individual’s performance in the second and first (SOA = 17 ms) TOJ task, henceforth noted ΔTOJ. ΔTOJ did not differ between the groups [F(1,28) = 0.27, p = 0.61, partial η^2^ = 0.01].) The BIS/BAS ‘reward responsiveness’ score and the ΔTOJ were taken as covariates in the analyses on participants’ performance at the subTOLI task.

#### Subthreshold temporal order cued luminance identification task

**Figure 4:**
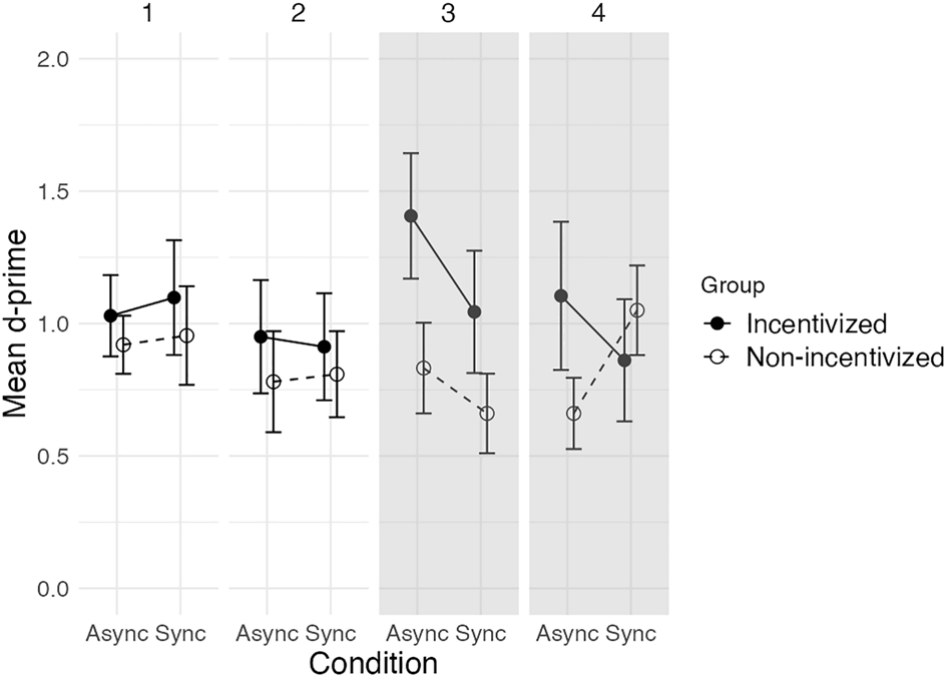
Mean d-prime values of the two experimental groups (Incentivized vs. Non-incentivized) in the Asynchronous (‘Async’) and Synchronous (‘Sync’) conditions in the 4 sub-blocks of the subthreshold temporal order cued luminance identification task. The Incentivized group received motivational instructions (*i.e.*, monetary incentive) between the 2nd and 3rd sub-blocks of the subthreshold version of the task, represented by the dashed line. The two groups differed by the presence/absence of motivational instructions in the 3rd and 4th sub-blocks (indicated with a gray background). Error bars represent ± SEM between participants.

To test whether the motivational instructions had an effect on the improvement in the Asynchronous condition, we conducted an ANCOVA on d-prime with condition (Asynchronous vs. Synchronous), block (‘before’ vs. ‘after motivational instructions’) and sub-block (1st vs. 2nd in the block before, and 3rd vs. 4th in the block after motivational instructions) as within-group variables, group (Incentivized vs. Non-incentivized) as a between-group variable, and ΔTOJ and BIS/BAS ‘reward responsiveness’ score as covariates. A significant three-way interaction was found between group, condition and block [F(1,26) = 4,24 p < 0.05, partial η^2^ = 0.14] (Fig. 4).

In order to decompose this three-way interaction, we calculated the difference in performance between the Asynchronous and Synchronous conditions (*i.e.,* d-prime_async_ – d-prime_sync_) for each participant and in each sub-block. We then conducted two separate ANCOVAs on this value in each block (separately for blocks before and after motivational instructions), with sub-block (1st vs. 2nd in the block before, and 3rd vs. 4th in the block after motivational instructions) as within-group variable, group (Incentivized vs. Non-incentivized) as a between-group variable, and ΔTOJ and BIS/BAS ‘reward responsiveness’ score as covariates. In the block after motivational instructions a main effect of group was found ([F(1,26) = 4.26, p < 0.05, partial η^2^ = 0.14]) with a larger positive performance difference in the Incentivized group (0.30) compared to the Non-incentivized group (-0.11).

As in Experiment 1, we checked whether our experimental manipulation affected participants’ decision criterion in addition to d-prime. We conducted an ANCOVA on the decision criterion with condition (Asynchronous vs. Synchronous), block (‘before’ vs. ‘after motivational instructions’) and sub-block (1st vs. 2nd and 3rd vs. 4th) as within-group variables, group (Incentivized vs. Non-incentivized) as a between-group variable, and ΔTOJ and BIS/BAS ‘reward responsiveness’ score as covariates. No significant main effect of group or interaction with group was found.

#### Suprathreshold temporal order cued luminance identification task

An ANOVA conducted on d-prime with condition (Asynchronous vs. Synchronous) as a within-group variable and group (Incentivized vs. Non-incentivized) as a between-group variable revealed a main effect of factor condition [F(1,28) = 44.16, p < 0.00001, partial η^2^ = 0.61], with a higher d-prime value in the Asynchronous (2.06) compared to the Synchronous condition (0.72). No main effect of factor group or interaction between factors was found indicating similar d-prime values in both groups.

An ANOVA conducted on beta with condition (Asynchronous vs. Synchronous) as a within-group variable and group (Incentivized vs. Non-incentivized) as a between-group variable revealed no significant main effect of group or interaction with group.

### Discussion of Experiment 2

Experiment 2 was designed to verify whether motivation played a role in the use of subthreshold order (SOA = 17 ms) to improve luminance identification. When participants received a monetary incentive motivating them to improve their performance by using the subthreshold temporal order cues to predict the targets’ luminance, a benefit linked to the use of subthreshold order was found in the Incentivized group but not in the Non-incentivized group. Results of the Non-incentivized group did not reveal any facilitation effect. The two groups did not differ in their baseline motivation levels as measured via self-questionnaires before the experiment, nor in their performance before the introduction of the monetary incentive. The reward responsiveness trait tended to be higher in the Non-incentivized group, but between-group differences persisted in the ANCOVA which took this variable as a covariate. Moreover, similar facilitation effects observed in both groups when order was suprathreshold (100 ms SOA), and similar performances in the subthreshold version of the task preceding the monetary incentive indicate that the two groups did not differ in their general ability to detect and use order to predict targets. Thus, the observed facilitation effect linked to the use of subthreshold order is likely due to our experimental manipulation of participants’ motivational levels via the monetary incentive. Additional analyses on order detection sensitivities in the subTOLI task compared to the TOJ task were consistent with these findings (see Supplementary Information S2).

## General discussion

The aim of the present study was to test the existence of automatic processing of temporal order, when the delay between sensory events is under the threshold for conscious temporal order perception. Our results suggest a facilitation effect on participants’ performance in the identification of target luminances when targets are preceded by a subthreshold order information compared to a control condition where the cues are synchronous and bear no predictive information. This replicates the facilitation effect described by Chassignolle et al.^16^ on RT and extends it to sensitivity (d-prime) in an experimental setup where targets are more difficult to identify. The main conclusion, however, is that the automatic processing of temporal order is very fragile, and sensitive to motivational effects.

Several aspects of our results showed the automatic processing of order to be fragile. In Experiment 1, the use of temporal order was transient at the beginning of the experiment and then its effect disappeared. In Experiment 2 it was present only in the Incentivized group, and only once participants had received motivational instructions. It should be noted that the facilitation effect observed at the beginning of the task (1st sub-block) in the Predictive group of Experiment 1 was not replicated in the Incentivized and Non-incentivized groups of Experiment 2, although the experimental conditions and instructions were identical at the beginning of the task in both experiments. We argue that this finding is consistent with the hypothesis that the facilitation effect linked to the processing and exploitation of subthreshold order is rather small and transitory. Analyses on decision criterion in both experiments revealed that performance cannot be explained by different response biases between groups.

Experiment 2 was designed to verify whether the processing of subthreshold temporal order was linked to motivation. The facilitation effect that was observed in Experiment 1 at the beginning of the task, but disappeared over the course of time, suggests that the effects resulted from the initial training in associating subthreshold order information to the target gray values, but this process is not purely automatic since the facilitation effect did not improve with time. Performance is known to decrease with time during monotonous tasks^28^, and a decrease of motivation or fatigue over time may explain the results. However, more direct evidence of a role of motivation was required. By manipulating participants’ motivational levels via a monetary incentive in Experiment 2, the facilitation effect in participants’ sensitivity was shown to depend on motivation. It has already been shown that top-down factors, like motivation, are necessary to establish predictive relations between stimuli even when the primers are presented at a subthreshold level^29,30^. Thus, the processing of subthreshold order may require such top-down factors to be initiated.

Since the motivational manipulation affects the processing of order, it is unlikely that the processing of order, as investigated in our study, is an automatic, permanent process in our day-to-day life. Even in the absence of motivation manipulation in Experiment 1, order processing occurred only after intensive training and wore off rapidly. This is logical, considering that if order processing were a continuously ongoing process, ordering events in time at a high temporal resolution would mean computational costs for the brain. As emphasized in the introduction, the perception of order is different from an order of percepts (*i.e.*, sequential processing of events). Processing the order means establishing a higher-level relationship between two events and encoding their relative position on the arrow of time. With more and more events unfolding and taking place as time passes, processing each event’s position relative to the ones already processed would quickly become a resource consuming activity for the brain. It is thus most likely that motivation is an important factor that serves as a promoter of temporal order processing at the level of automatic and subthreshold visual information processing, when such process is needed or can bring a potential benefit in the task at hand.

One might wonder whether the fragile effects observed in the present study really concerned order processing, or only the asynchrony between the stimuli. Several control conditions allow us to discard alternative possibilities. In Experiment 1, we manipulated the predictiveness of the cue order in two groups (Predictive vs. Non-predictive group). This experimental manipulation allows us to rule out the possibility that the simple asynchrony between the cues (and not specifically their order) could have an effect on sensitivity. Since the facilitation effect was only observed in the Predictive group, this alternative explanation can be ruled out.

Additionally, we verified that the observed effects reflect a facilitation due to subthreshold order processing and not simply responses to the mere order information. To do this, we checked whether participants answered to the temporal order of the cues rather than to the target luminances, by comparing responses according to the response side, and according to the congruency between temporal order and responses (see Supplementary Information S1), but these factors could not explain our results.

Taken together, these results suggest that the processing of temporal order of sub-threshold asynchronies is possible, although it likely depends on task requirements.

It is remarkable that the effect was fragile even though participants were explicitly informed about the contingency between order and target luminances. Whether it is possible to process subthreshold order when no attention is drawn to order-target contingencies seems unlikely in light of our results, however it may be possible with other manipulations of top-down, possibly attentional factors^30,31^. Future studies investigating the automaticity of order processing may focus their design on similar experiments to ours, but where order is presented supraliminally, and no explicit instructions mention its contingency with target stimuli.

## Supplementary Information

Supplementary Information.

## Data Availability

The datasets generated during and/or analyzed during the current study are available from the corresponding author on reasonable request.
