# Supplementary material for: The processing of subthreshold visual temporal order is transitory and motivation-dependent
Source: Sci Rep. 2023 May 11;13:7699. doi: 10.1038/s41598-023-34392-5 (PMC10175535; doi:10.1038/s41598-023-34392-5)
Supplement: Supplementary file 1 — Supplementary Information. [file 41598_2023_34392_MOESM1_ESM.pdf]

The processing of subthreshold visual temporal order is transitory and motivation-dependent

Patrik Polgári[1], Ljubica Jovanovic[1], Virginie van Wassenhove[2], and Anne Giersch[1,3]*

[1] University of Strasbourg, INSERM U1114, Strasbourg, France
[2] Cognitive Neuroimaging Unit, CEA, INSERM, CNRS, Neurospin, Université Paris-Saclay, F-91191 Gif/Yvette, France
[3] Department of Psychiatry, University Hospital of Strasbourg, Strasbourg, France

* Corresponding author: Anne Giersch
Department of Psychiatry, University Hospital of Strasbourg, 1, pl de l'Hôpital, 67000 Strasbourg, France
email: giersch@unistra.fr
tel: +33-388-116-471

S1: Verifying the potential confounding effect of responding to the order of the cues rather than the target luminances

In order to exclude potential confounding factors that could explain our results, we verified a scenario in which participants' responses do not depend on their luminance discrimination abilities, but on their order judgment. In this case, if responses were made to the order of the cues rather than the target luminances, as in a classical temporal order judgment task, participants' performances at our task (i.e., luminance discrimination performance) would be biased depending on the side of response relative to the order of presentation of the cues (correct response corresponding to the side of 1st stimulus or 2nd stimulus, e.g., correct response to luminance -light or dark- on the Left when cues appear in Left-Right order, and correct response to luminance -light or dark- on the Right when cues appear in Right-Left order).

We checked this possibility by conducting repeated measures ANOVAs, performed separately in the Predictive and Non-predictive group, on participants' d-prime value with condition (Asynchronous vs. Synchronous) and sub-block (1st vs. 2nd vs. 3rd vs. 4th) as within-group variables, and side of response (response to the side of the 1st vs. the 2nd cue stimulus) as a between-group variable. The variable side of response had no significant effect ([$F(1,10)=0.28$, $p>0.05$, partial $\eta^2=0.03$] for the Predictive group; [$F(1,10)=0.26$, $p>0.05$, partial $\eta^2=0.03$] for the Non-predictive group), nor did it interact with the other two variables in either of the groups (all p-values > 0.05), indicating no response bias linked to the side of response (in the overall task or in any specific sub-block).

If participants responded to the order of the cue stimuli, another implication would be that in trials where the order of the cues does not predict correctly the luminance (i.e., in 50% of trials in the Non-Predictive group) responses that follow the order of the cues as participants were trained would be counted as errors. This would mean a higher error rate in incongruent Asynchronous trials (i.e., incongruency between cue order and target luminance) compared to congruent Asynchronous trials (i.e., congruency between cue order and target luminance). To verify this possibility, we calculated the correct response rate separately for incongruent and congruent Asynchronous trials in all 4 sub-blocks in the Non-predictive group. (Note that in the Predictive group all trials were congruent, so this analysis was not possible). A repeated measure ANOVA conducted on the correct response rate with sub-block (1st vs. 2nd vs. 3rd vs. 4th) and congruency (incongruent vs. congruent) as within-group variables revealed no effect of congruency [$F(1,11)=0.32$, $p>0.05$, partial $\eta^2=0.03$], or interaction between congruency and sub-block [$F(3,33)=0.90$, $p>0.05$, partial $\eta^2=0.08$], indicating that performance was not biased

toward the order of the cues in incongruent trials and thus was not lower in incongruent trials compared to congruent trials in either of the sub-blocks.

S2: Verification of results by comparing order detection sensitivities (d-prime) in TOJ and subTOLI tasks

Following Meyen et al.'s (2022) study, we computed d-prime as a sensitivity measure of order detection for each participant in the two TOJ tasks separately (our "direct task") and took the d-prime measure from the Asynchronous trials in the sub-blocks of the subTOLI task in which we found significant effects (our "indirect task"). We considered these d-prime values as valid since their calculation is based on a similar number of trials. If any there was more trials in the TOJ task than in the subTOLI one: 24 asynchronous and 24 synchronous in the subTOLI task, 40 trials per SOA in the TOJ task). Hence, the number of participants was the same for both tasks.

Experiment 1:

For the subTOLI task, d-prime values of the Predictive group were the same as in the analyses in the main manuscript since for them order and luminance levels were congruent. D-prime values were computed for the Non-predictive group based on the order of the cues: we considered a response as correct if it corresponded to the contingency learned during training.

First, we performed an ANOVA comparing d-prime in the first TOJ task (performed at the beginning of the experiment) and the first sub-block of the subTOLI task (the sub-block in which we found significant effects and performed right after the first TOJ task and the training blocks). Due to their temporal proximity at the beginning of the experiment we considered that the first subTOLI sub-block is more comparable to the first TOJ task than the second. An interaction between group and task was found [$F(1,22)= 18.29$, $p<0.0005$, partial $\eta^2=0.45$]. Sub-analyses showed that for the Predictive group d-prime in the subTOLI task (1.68) was significantly higher compared to the TOJ task (0.21) [$F(1,22)=17.7$, $p<0.001$, partial $\eta^2=0.45$]. This difference in d-prime values between the subTOLI and TOJ tasks (0.17 vs. 0.11, respectively) was not significant in the Non-predictive group [$F(1,22)=0.12$, $p>0.05$, partial $\eta^2=0.01$].

We verified these effects when only considering trials for the Non-predictive group with the same order-luminance level congruency that was learned during the training (i.e., half of Asynchronous trials). This time, no interaction was found, however the main effect of task was

significant [$F(1,22)= 38.43$, $p<0.001$, partial $\eta^2=0.64$]. Sub-analyses showed that d-prime values in both groups were significantly higher in the subTOLI task compared to the TOJ task (Predictive group: see paragraph above; Non-predictive group: [$F(1,22)= 12.0$, $p<0.005$, partial $\eta^2= 0.35$], d-prime(subTOLI) = 1.04, d-prime(TOJ) = 0.11).

Additionally, in the Non-predictive group, we compared d-prime values as a function of the correspondence of order-luminance level congruency to training (same vs. opposite to training). A significant difference was found [$F(1,11)=24.47$ $p<0.0005$, partial $\eta^2=0.69$] with higher d-prime values for trials where the congruency was the same as during training (1.04) compared to trials with the opposite congruency (-0.68).

Experiment 2:

For the subTOLI task d-prime values were the same as in the analyses in the main manuscript since in Experiment 2 order and luminance levels were congruent in 100% of trials for both groups. We verified our main results in Experiment 2 by performing an ANOVA comparing d-prime in the 3rd sub-block of the subTOLI task and the 2nd TOJ tasks (since both were performed in the second half of the experiment and after the monetary incentivization given to the Incentivized group). Again, we considered these d-prime values as valid since their calculation is based on a similar number of trials, which remained the same as in Experiment 1. The number of participants was also the same for the TOJ and subTOLI tasks. A significant main effect of task [$F(1,28)= 36.25$, $p<0.000005$, partial $\eta^2=0.56$] and an interaction between factors group and task was found [$F(1,28)= 7.75$, $p<0.05$, partial $\eta^2= 0.22$]. Sub-analyses showed that both groups had a higher d-prime value in the subTOLI task compared to the d-prime in the TOJ task (Incentivized group: [$F(1,14)= 28.7$, $p<0.0005$, partial $\eta^2=0.67$], d-prime(subTOLI) = 1.41, d-prime(TOJ) = -0.05; Non-incentivized group: [$F(1,14)= 8.08$, $p<0.05$, partial $\eta^2=0.37$], d-prime(subTOLI) = 0.83, d-prime(TOJ) = 0.30) indicating higher subthreshold order discrimination sensitivity in the indirect task compared to the direct task. Further sub-analyses indicated that the difference in d-prime values between the subTOLI and TOJ tasks was larger in the Incentivized group than in the Non-incentivized group, due to higher d-prime values in the TOJ task for the Non-incentivized group compared to the Incentivized group [$F(1,28)= 6.00$, $p<0.05$, partial $\eta^2=0.18$].

The comparison of the 4th sub-block of the sub-TOLI and the 2nd TOJ tasks showed a significant main effect of task [$F(1,28)= 23.71$, $p< 0.00005$, partial $\eta^2=0.46$] and an interaction between factors group and task [$F(1,28)= 6.41$, $p<0.05$, partial $\eta^2= 0.19$]. Sub-analyses showed that the Incentivized group had a higher d-prime value in the subTOLI task compared to the d-

prime in the TOJ task ([$F(1,14)= 17.9$, $p<0.005$, partial $\eta^2= 0.56$], d-prime(subTOLI) = 1.10, d-prime(TOJ) = -0.05) indicating higher subthreshold order discrimination sensitivity in the indirect task compared to the direct task. After correction for multiple comparisons this difference was not significant in the Non-incentivized group [$F(1,14)= 5.80$, $p=0.06$, partial $\eta^2=0.29$] (d-prime(subTOLI) = 0.66, d-prime(TOJ) = 0.30). In the 4th sub-block of the subTOLI task d-prime values did not differ between the groups.

We ran the same ANOVAs on blocks of the first half of the experiment (1st subTOLI sub-block vs. 1st TOJ task, and 2nd subTOLI sub-block vs. 1st TOJ task). In both analyses we found a main effect of task with higher d-prime value averaged over groups in the respective sub-block of the subTOLI task compared to the 1st TOJ task indicating higher subthreshold order discrimination sensitivity in the indirect task compared to the direct task in both groups (sub-analysis with sub-block 1: [$F(1,28)= 30.40$, $p< 000001$, partial $\eta^2= 0.52$], mean d-prime(subTOLI) = 0.97, mean d-prime(TOJ) = 0.31; sub-analysis with sub-block 2: [$F(1,28)=12.03$, $p<0.005$, partial $\eta^2=0.30$], mean d-prime(subTOLI) = 0.87, mean d-prime(TOJ) = 0.31).

The main effect of task found throughout the ‘subTOLI sub-block vs. TOJ’ comparisons indicates that both groups had higher subthreshold order discrimination sensitivity in the indirect task compared to the direct (TOJ) task, but this d-prime difference was larger in the Incentivized group after monetary incentive was given to this group (3rd and 4th sub-blocks). These results are in line with our main findings and interpretations described in the main manuscript. Of course, we cannot exclude the possibility that monetary incentives had an effect on the conscious perception of order cues separated by 17 ms during the main task (i.e., lowering of perceptual threshold) which would similarly result in increased d-prime values in the Incentivized group in the subTOLI task. In this case, order could be detected consciously at least in some trials, and used to predict the target luminance levels. This however makes our argument about the fragility of order detection and its use even stronger: the exploitation of order information is still difficult and transitory, as the facilitation effect disappeared in the next sub-block.

References

Meyen, S., Zerweck, I. A., Amado, C., von Luxburg, U., & Franz, V. H. (2022). Advancing research on unconscious priming: When can scientists claim an indirect task advantage? Journal of Experimental Psychology: General, 151(1), 65–81. https://doi.org/10.1037/xge0001065
